# Effect and Removal Mechanisms of 6 Different Washing Agents for Building Wastes Containing Chromium

**DOI:** 10.1100/2012/298407

**Published:** 2012-07-31

**Authors:** Wang Xing-run, Zhang Yan-xia, Wang Qi, Shu Jian-min

**Affiliations:** ^1^State Key Laboratory of Environmental Criteria and Risk Assessment, Chinese Research Academy of Environmental Sciences, Beijing 100012, China; ^2^College of Science, Northwest A&F University, Shaanxi 712100, China

## Abstract

With the building wastes contaminated by chromium in Haibei Chemical Plan in China as objects, we studied the contents of total Cr and Cr (VI) of different sizes, analyzed the effect of 6 different washing agents, discussed the removal mechanisms of 6 different washing agents for Cr in various forms, and finally selected applicable washing agent. As per the results, particle size had little impact on the contents of total Cr and Cr (VI); after one washing with water, the removal rate of total Cr and Cr (VI) was 75% and 78%, respectively, and after the second washing with 6 agents, the removal rate of citric acid was the highest, above 90% for total Cr and above 99% for hexavalent chromium; the pH of building wastes were reduced by citric acid, and under acid condition, hexavalent chromium was reduced to trivalent chromium spontaneously by organic acid, which led to better removal rate of acid soluble Cr and reducible Cr; due to the complexing action, citric acid had best removal rate for oxidizable trivalent chromium. In conclusion, citric acid is the most applicable second washing agent for building wastes.

## 1. Introduction

So far, there are 75 chromate enterprises in China. Due to the small scale, the backward techniques, and high environmental pollution of chromate enterprises in China, over 50 enterprises have been shut down. Not only the soil around the closed chromate enterprises had been severely contaminated, but there were serious environmental pollutions in the building wastes [1, 2]. In Minfeng Chemical Plant in Chongqing, the contaminated building wastes were about 30,000 m^3^ and the content of hexavalent chromium was 2374 mg/kg in building wastes; in Haibei Chemical Plant in Qinghai Province, there were at least 7000 m^3^ building wastes from sintering workshop and leaching workshop, and the content of hexavalent chromium in concrete of internal foundation was up to 6278 mg/kg. Due to the different composition of building wastes and soil, and the different forms of chromium occurrence in building wastes and soil the applicable treatment technologies for soil are not suitable for building wastes. As the progress of chromium contaminated soil remediation all over all China, the treatment of building wastes is of great urgency.

In abroad, studies on contaminated soil remediation have been started for some time, and large amount of works have been done in Europe, USA and Japan [3–5]. Chemical washing can be used to separate and isolate hazardous substances or turn hazardous substances harmless [6], and besides, the technique is suitable for wide use based on its advantages, such as low energy consumption, low equipment investment, wide application scope and quick effect. The key point of chemical washing is to select and develop washing agents [7]. The commonly used washing agents includes water, acid [8], saline solution, chelating agent [9], surfactant [10], and so forth. The most economic and environmental protection washing agent is water, and one chromium-plating company in USA, United Chromium (Corvallis, OR), was using water to washing Cr (VI) in project site, which reduced the concentration of chromium from 1923 mg/kg to 65 mg/kg [11]. EDTA is also one of the commonly used washing agent and it can generate stable chelating agent by reacting with most metals [12]. As indicated by Tampouris [13], the removal rate of Zn and Cd in contaminated soil by HCl + CaCl_2_ was 78% and 70%, respectively. Lee et al. [14] found that the removal rate of As in sand at river bottom was 95% by citric acid washing. Bhattacharya et al. [15] adopted oxalates to remove the Cr in soil from lumber yard and 98% Cr was removed. However, there are few studies on the treatment of building wastes contaminated by chromium.

In this study, it adopted deionized water, EDTA, citric acid, oxalic acid, HCl, and acetic acid as washing agents to compare the removal effect of Cr in building wastes contaminated by Cr and select applicable agent and analyzed the removal mechanism of those 6 washing agents with the hope of providing technical supports for the disposal of building wastes contaminated by chromium.

## 2. Materials and Methods

### 2.1. Chemicals and Reagents

The concrete paved inside the chromate production workshop in Beihai Chromate Plant was severely contaminated by Cr; the study took the concrete as object and sampled at 5 different locations; the collected concretes were dried in the air naturally and samples of 5 different sizes were fabricated by the crushing with 20, 50, 80, 100, and 150 mesh screens; the particle sizes were 0.31–0.87 mm, 0.19–0.31 mm, 0.15–0.19 mm, 0.11–0.15 mm and <0.11 mm; samples of the same size were mixed uniformly and stored properly for later use. Samples of 5 different sizes were mixed at 1 : 1 mass ratio and their physicochemical properties are shown in Table 1. Washing agent: deionized water, 0.05 mol/L and 0.1 mol/L EDTA-Na_2_, 0.1 mol/L and 0.5 mol/L citric acid, 0.1 mol/L and 0.5 mol/L oxalic acid, 0.1 mol/L, 0.5 mol/L and 1 mol/L HCl, and 0.1 mol/L and 0.5 mol/L acetic acid.

### 2.2. Testing Methods

Accurately weight 5.00 g sample and place it in a 250 mL conical flash, add 50 mL washing agent in the flash with solid-to-liquid ratio of 1 : 10 (i.e., 1 g : 10 mL), wash 40 min with electromagnetic stirring, and filter the mixture to collect eluent and solid sample. The washed sample shall be dried and properly stored for future use.

### 2.3. Sample Analysis

Alkaline digestion [16] was applied to dissolve the Cr (VI) in solid sample, and atomic absorption spectrophotometry was used to test the concentration of Cr (VI) in dissolution. Microwave-assisted acid digestion [17] was used to dissolve the total Cr in solid sample, and atomic absorption spectrophotometry was used to test the concentration of total Cr in dissolution. Coprecipitation was used to dissolve the Cr (VI) in eluent and atomic absorption spectrophotometry was used to test the concentration of Cr (VI). Modified BCR sequential extraction was adopted to extract the soluble, acid soluble, reducible, oxidizable, and residual Cr in solid sample while atomic absorption spectrophotometry was used to test the concentration of Cr in extract.

## 3. Results and Discussion

### 3.1. Impact of Particle Sizes

#### 3.1.1. Impact on Cr Content in Different Sizes

**Figure 1:**
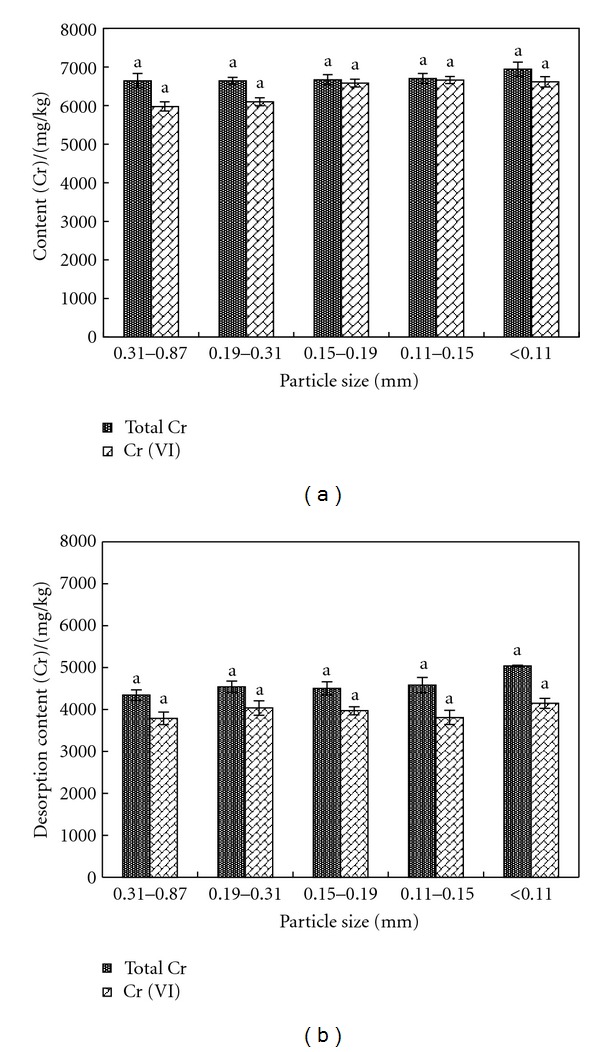
Contents and desorption contents of total Cr and Cr (VI) in 5 samples of different sizes.

The contents of total Cr and Cr (VI) in 5 samples of different sizes were measured, as shown in Figure 1(a). After being washed 40 min in deionized water, the desorption contents of total Cr and Cr (VI) in 5 samples were measured, as shown in Figure 1(b).

As indicated by Figure 1(a), the content of total Cr in sample was around 6600 mg/kg and the content of Cr (VI) was around 6300 mg/kg. Figure 1(a) also showed that there was no distinct difference on the contents of total Cr and Cr (VI) in different samples. As indicated by Figure 1(b), there was no distinct difference on the desorption contents of total Cr and Cr (VI) in 5 samples. Thus, particle size had no distinct impact either on the contents of total Cr and Cr (VI) in samples or on the desorption contents of total Cr and Cr (VI). Therefore, in the actual disposal of building wastes contaminated by Cr, it is not necessary to consider the impact of particle size and from the aspect of crushing cost, it shall select more economic crushing equipments. In following experiments, the study adopted 20 mesh screen after crushing.

#### 3.1.2. Impact on Cr Forms in Different Sizes

**Figure 2:**
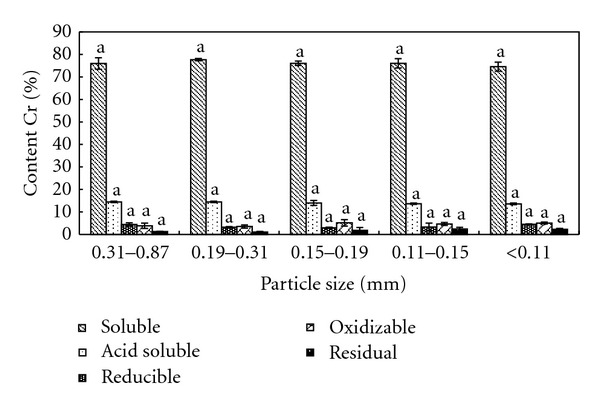
Different forms of Cr in 5 samples of different sizes.

The contents of Cr in different forms in samples of different sizes were tested, as shown in Figure 2. As indicated by Figure 2, for Cr in different forms in sample of same size, the major form of Cr was soluble, about 75% of the total, and the percentages of acid soluble, reducible, oxidizable, and residual Cr were 15%, 4%, 4% and 2%, respectively. For Cr of same form in different samples, there was no distinct difference on their contents, indicating that particle size had no distinct impact on the contents of Cr in different forms in samples.

### 3.2. Comparison of Washing Effects by Different Agents

#### 3.2.1. Effect of Water Washing

**Figure 3:**
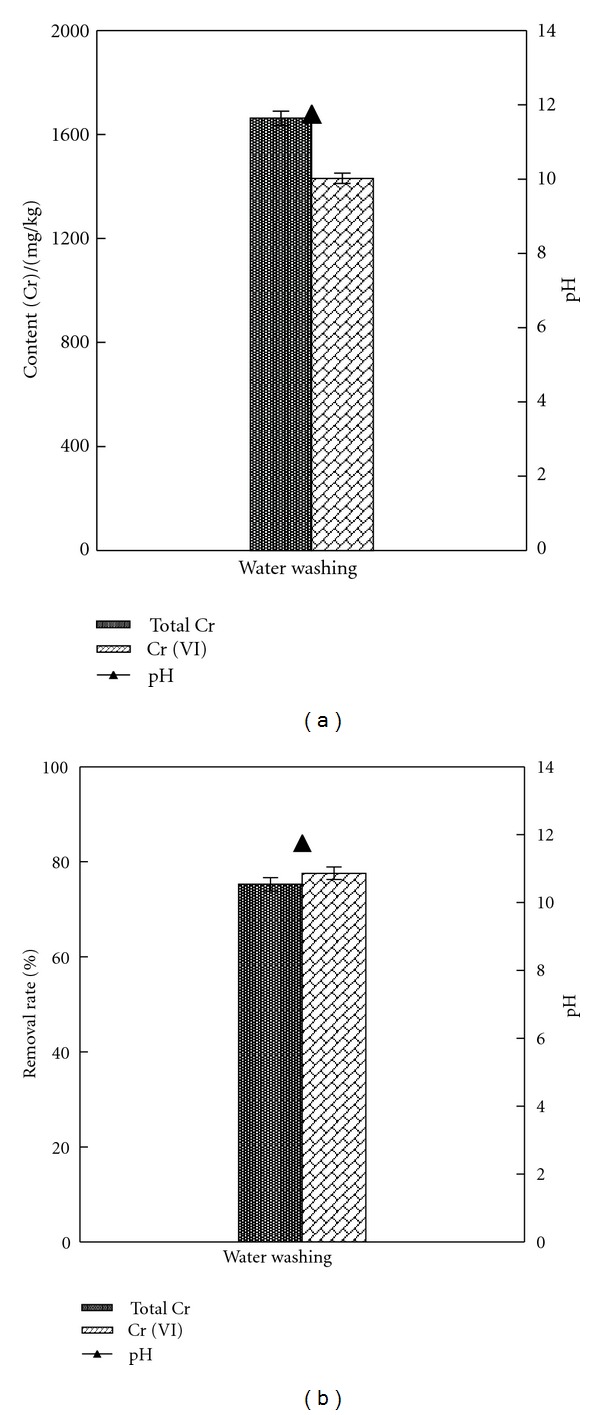
Content of total Cr and Cr (VI) and the pH in solid sample after water washing.

The content of soluble Cr was high in sample, about 75%. Thus, it adopted washing by deionized water firstly and then tested the contents of total Cr and Cr (VI) in solid sample, as shown by Figures 3(a) and 3(b). The result showed that the remained content of total Cr and Cr (VI) after water washing was 1662.25 mg/kg and 1431.40 mg/kg while the removal rate of total Cr and Cr (VI) was 75.24% and 77.59%, respectively.

 As shown by Table 2, the pH of sample was 11.74 after water washing, which was strongly alkaline. Cr (III) was mainly in the form of positive ions, such as Cr^3+^, Cr(OH)^2+^, and Cr(OH)^2+^, and under alkaline conditions, it was hard for the hydroxides to dissolve in water. Cr (VI) was mainly in the form of negative ions, such as CrO_4_^2−^, HCr_2_O_7_^2−^, HCrO_4_^3−^, and Cr_2_O_7_^2−^, and it was easy for sodium, potassium, and ammonium salt to dissolve in water. Thus, it was mainly to remove Cr (VI) in water washing. Although the removal rate reached 75% after water washing, the content of Cr in sample was still high, and it was still quite harmful and needed further disposal by second washing.

#### 3.2.2. Effect of Second Washing by Different Agent

**Figure 4:**
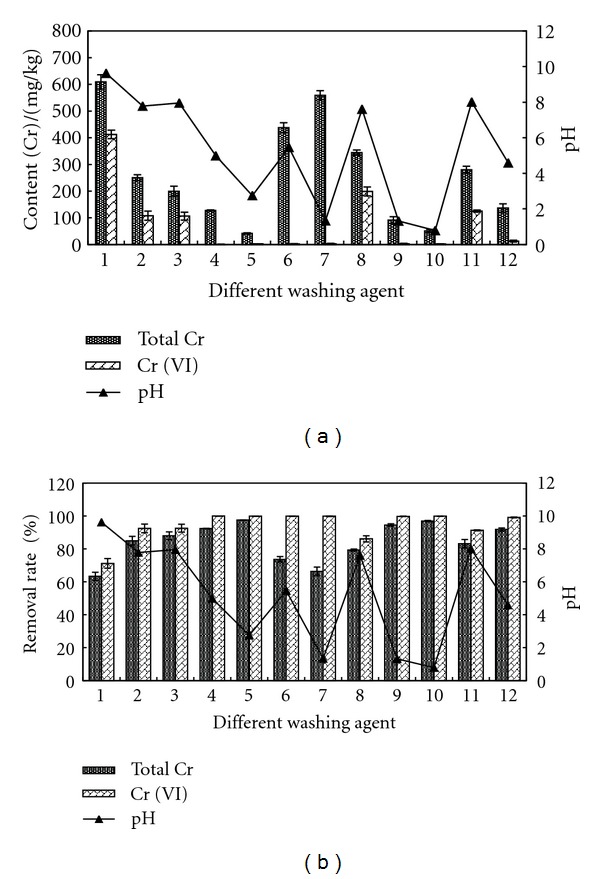
Comparison of total Cr, Cr (VI), and pH in solid sample after being washed by different agents. Washing agent: 1—water; 2—0.05 mol/L EDTA-Na_2_; 3—0.1 mol/L EDTA-Na_2_; 4—0.1 mol/L citric acid; 5—0.5 mol/L citric acid; 6—0.1 mol/L oxalic acid; 7—0.5 mol/L oxalic acid; 8—0.1 mol/L HCl; 9—0.5 mol/L HCl; 10—1 mol/L HCl; 11—0.1 mol/L acetic acid; 12—0.5 mol/L acetic acid.

After water washing, second washing was performed with different agents. The contents of total Cr and Cr (VI) after washing were tested, as shown in Figure 4. The result showed that there were distinct differences on the remained contents and the removal rates of total Cr and Cr (VI). The removal effects of total Cr by citric acid, concentrated HCl, and concentrated acetic acid were better, and the removal rates were all above 90%. The contents of remained total Cr were 41.5–136.67 mg/kg. The removal effects of Cr (VI) by citric acid, oxalic acid, concentrated HCl, and concentrated acetic acid were better, and the removal rates were above 99%. The contents of remained Cr (VI) were 0.96–12.66 mg/kg. Through comparison, we can see that the removal effect of citric acid was best.

#### 3.2.3. Total Cr and Cr (VI) Content Variation

**Figure 5:**
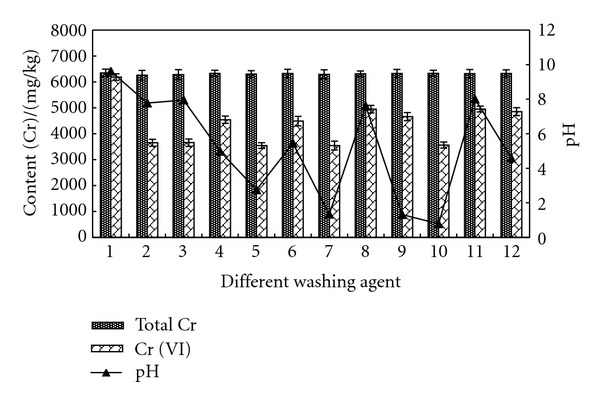
Total Cr and Cr (VI) content variation before and after washing. Washing agent: 1—water; 2—0.05 mol/L EDTA-Na_2_; 3—0.1 mol/L EDTA-Na_2_; 4—0.1 mol/L citric acid; 5—0.5 mol/L citric acid; 6—0.1 mol/L oxalic acid; 7—0.5 mol/L oxalic acid; 8—0.1 mol/L HCl; 9—0.5 mol/L HCl; 10—1 mol/L HCl; 11—0.1 mol/L acetic acid; 12—0.5 mol/L acetic acid.

Test the contents of total Cr and Cr (VI) in solid sample and eluent after second washing, and calculate the total contents of Cr and Cr (VI) by adding the two values, as shown in Figure 5.

**Figure 6:**
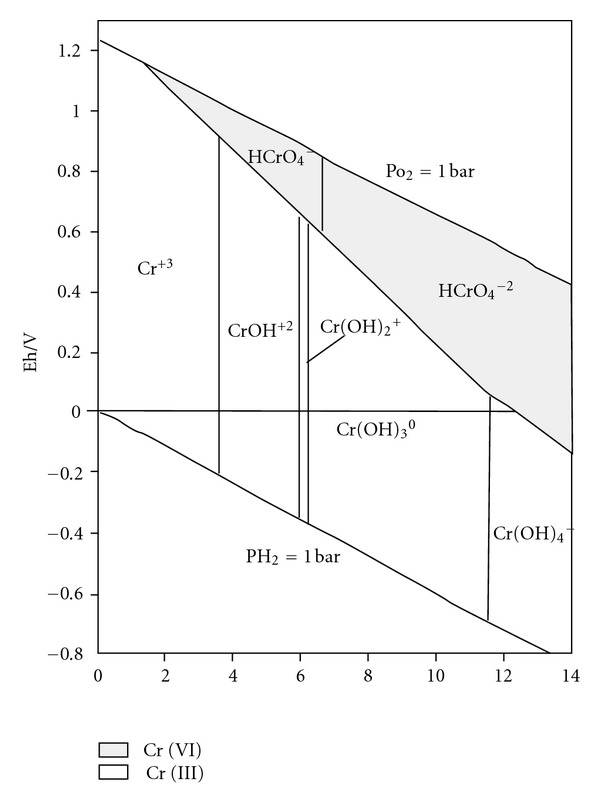
Eh-pH of Cr ion forms (25°C).

As indicated in Figure 5, the contents of total Cr remained the same, about 6600 mg/kg, and the contents of Cr (VI) was reduced, indicating that Cr (VI) was reduced into Cr (III). The pH of citric acid, oxalic acid, HCl, and acetic acid eluent was <6 and under acidic condition, the oxidation-reduction potential was larger than 0 and Cr (VI) was reduced into Cr (III), which reacted spontaneously, as shown in Figure 6. Thus, citric acid, oxalic acid, HCl, and acetic acid can remove Cr (VI) by both dissolution and reduction.

### 3.3. Removal of Soluble Cr

**Figure 7:**
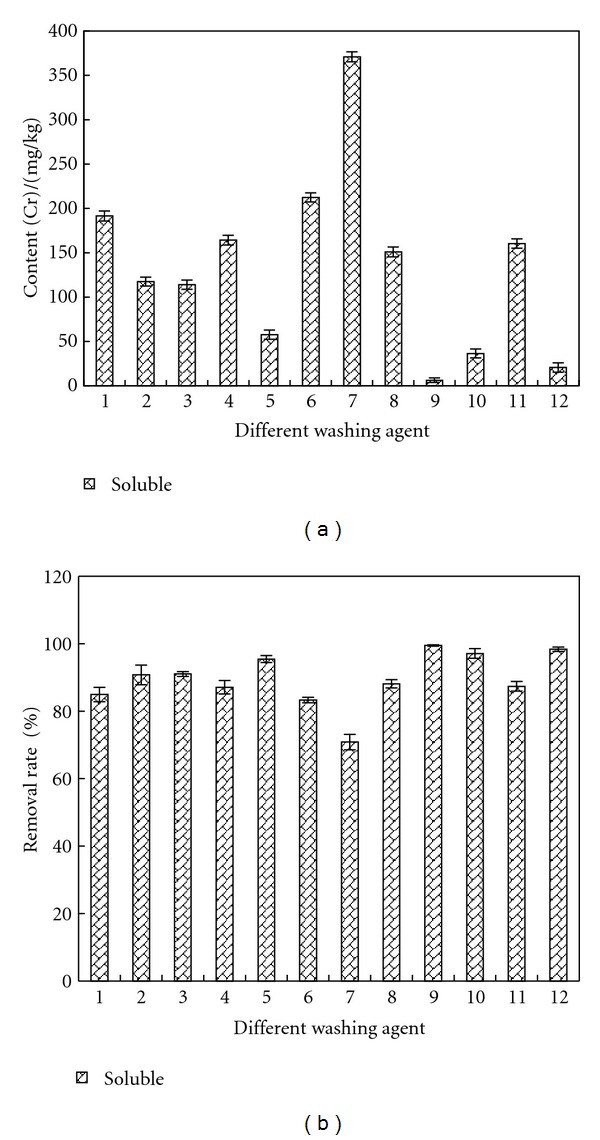
Removal effect of soluble Cr by different washing agents. Washing agent: 1—water; 2—0.05 mol/L EDTA-Na_2_; 3—0.1 mol/L EDTA-Na_2_; 4—0.1 mol/L citric acid; 5—0.5 mol/L citric acid; 6—0.1 mol/L oxalic acid; 7—0.5 mol/L oxalic acid; 8—0.1 mol/L HCl; 9—0.5 mol/L HCl; 10—1 mol/L HCl; 11—0.1 mol/L acetic acid; 12—0.5 mol/L acetic acid.

Figure 7 shows the remained contents and removal rates of soluble Cr in sample after being washed by different agents. As indicated by the results, the second washing of deionized water reduced the content of soluble Cr from 1072.85 mg/kg to 193.34 mg/kg, and the removal rate was 83%. The best removal agents for soluble Cr are concentrated citric acid, concentrated hydrochloric acid, and concentrated acetic acid. With above washing agents, the remained contents of soluble Cr in sample were below 50 mg/kg, and the removal rates were all above 95%.

In sample, Cr (VI) is mainly in soluble form and the reason that concentrated citric acid, concentrated hydrochloric acid, and concentrated acetic acid can obtain better removal effect is that they cannot only dissolve the soluble Cr contained by sample in water, but reduce it into Cr (III) of other forms, which decreases the amount of soluble Cr.

### 3.4. Removal of Acid Soluble Cr

**Figure 8:**
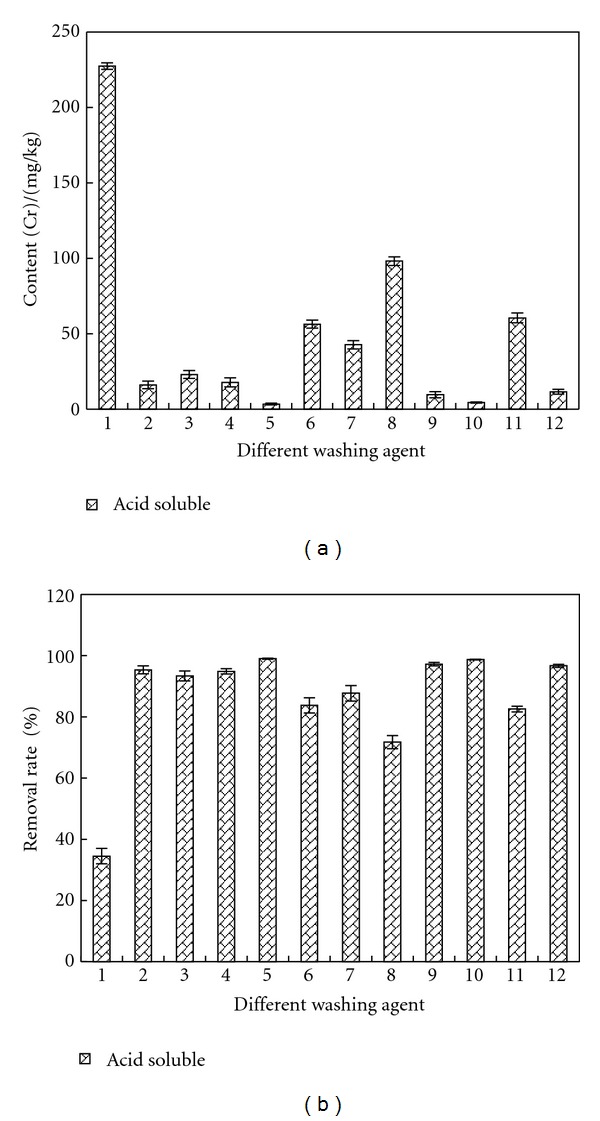
Removal effect of acid soluble Cr by different washing agents. Washing agent: 1—water; 2—0.05 mol/L EDTA-Na_2_; 3—0.1 mol/L EDTA-Na_2_; 4—0.1 mol/L citric acid; 5—0.5 mol/L citric acid; 6—0.1 mol/L oxalic acid; 7—0.5 mol/L oxalic acid; 8—0.1 mol/L HCl; 9—0.5 mol/L HCl; 10—1 mol/L HCl; 11—0.1 mol/L acetic acid; 12—0.5 mol/L acetic acid.

Figure 8 shows the remained contents and removal rates of acid soluble Cr in sample after being washed by different agents. The content of acid soluble Cr was reduced from 347.25 mg/kg to 227.41 mg/kg by deionized water and the removal rate was 34.5%. The removal rate of EDTA was 93.36–95.35%, that of citric acid was 94.86–99.00%, that of oxalic acid was 83.76–87.70%, that of HCl was 71.75–98.72%, and that of acetic acid was 82.56–96.67%. As indicated by Figure 4, the addition of above agents can reduce the pH of sample, and under acidic conditions, the removal rate of acid soluble Cr was better by all the previous agents.

### 3.5. Removal of Reducible Cr

**Figure 9:**
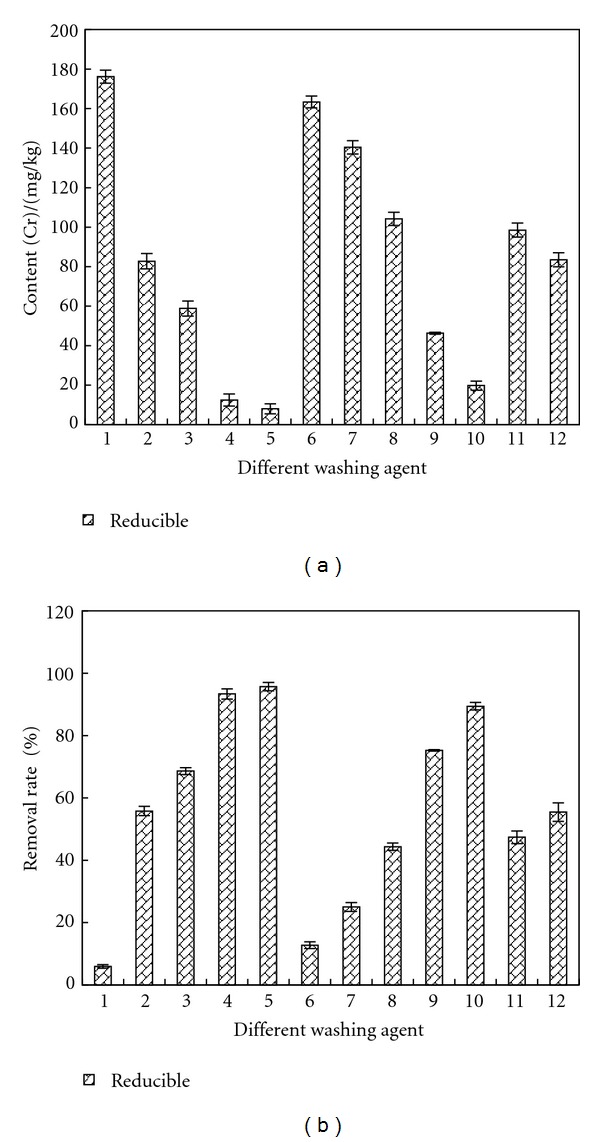
Removal effect of reducible Cr by different washing agents. Washing agent: 1—water; 2—0.05 mol/L EDTA-Na_2_; 3—0.1 mol/L EDTA-Na_2_; 4—0.1 mol/L citric acid; 5—0.5 mol/L citric acid; 6—0.1 mol/L oxalic acid; 7—0.5 mol/L oxalic acid; 8—0.1 mol/L HCl; 9—0.5 mol/L HCl; 10—1 mol/L HCl; 11—0.1 mol/L acetic acid; 12—0.5 mol/L acetic acid.

Figure 9 shows the remained contents and removal rates of reducible Cr in sample after being washed by different agents. The content of reducible Cr was reduced from 187.24 mg/kg to 176.17 mg/kg by deionized water and the removal rate was 5.91%. The removal effects of citric acid and concentrated HCl were better with removal rate of 93.33% and 95.71%, respectively. After being washed by citric acid and concentrated HCl, the ramained content of reducible Cr in sample was 12.49 mg/kg and 8.04 mg/kg.

Reducible Cr was mainly made up of Cr (VI). Citric acid and concentrated HCl can reduce the pH of sample and under acidic condition, the oxidation-reduction potential was larger than 0 and Cr (VI) was reduced into Cr (III), which reacted spontaneously, which led to the better removal effect of reducible Cr. The add of citric improved the content of organic acid in samples, which assisted Cr (VI) being reduced into Cr (III).

### 3.6. Removal of Oxidizable Cr

**Figure 10:**
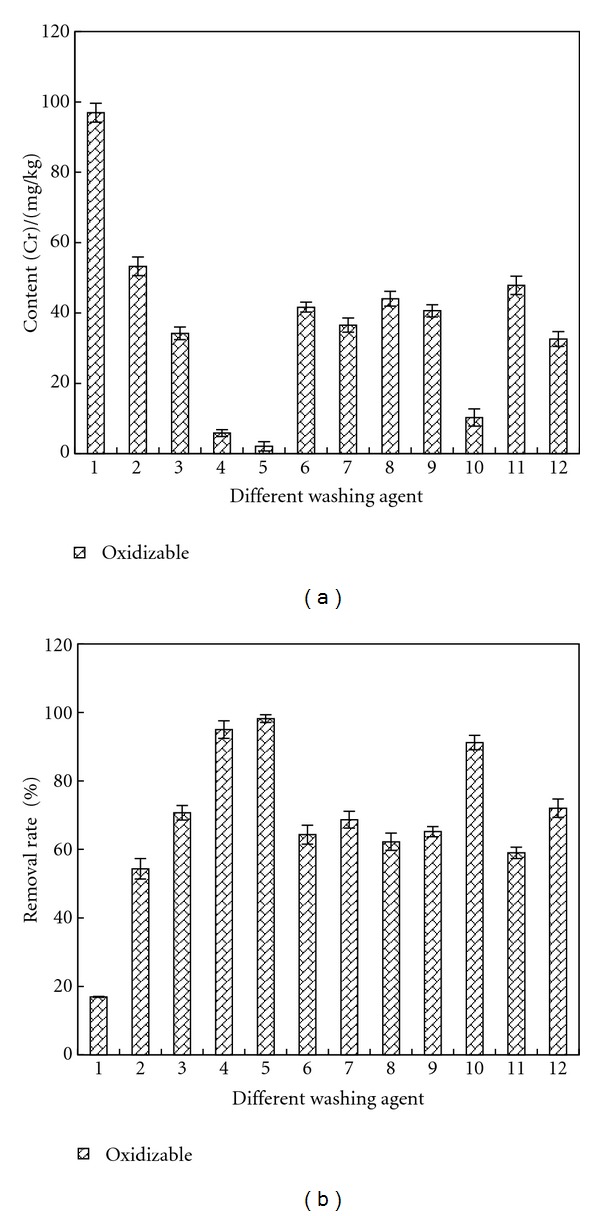
Removal effect of oxidizable Cr by different washing agents. Washing agent: 1—water; 2—0.05 mol/L EDTA-Na_2_; 3—0.1 mol/L EDTA-Na_2_; 4—0.1 mol/L citric acid; 5—0.5 mol/L citric acid; 6—0.1 mol/L oxalic acid; 7—0.5 mol/L oxalic acid; 8—0.1 mol/L HCl; 9—0.5 mol/L HCl; 10—1 mol/L HCl; 11—0.1 mol/L acetic acid; 12—0.5 mol/L acetic acid.

Figure 10 shows the remained contents and removal rates of oxidizable Cr in sample after being washed by different agents. The disposal effect of citric acid was the best, reducing the content of oxidizable Cr from 156.68 mg/kg to 10 mg/kg, and the removal rate was over 95%. Oxidizable Cr was mainly made up of Cr (III). Due to complexing action [18], EDTA, citric acid, oxalic acid, and acetic acid can better remove oxidizable Cr, especially citric acid, and the removal rates were all above 60%.

### 3.7. Removal of Residual Cr

**Figure 11:**
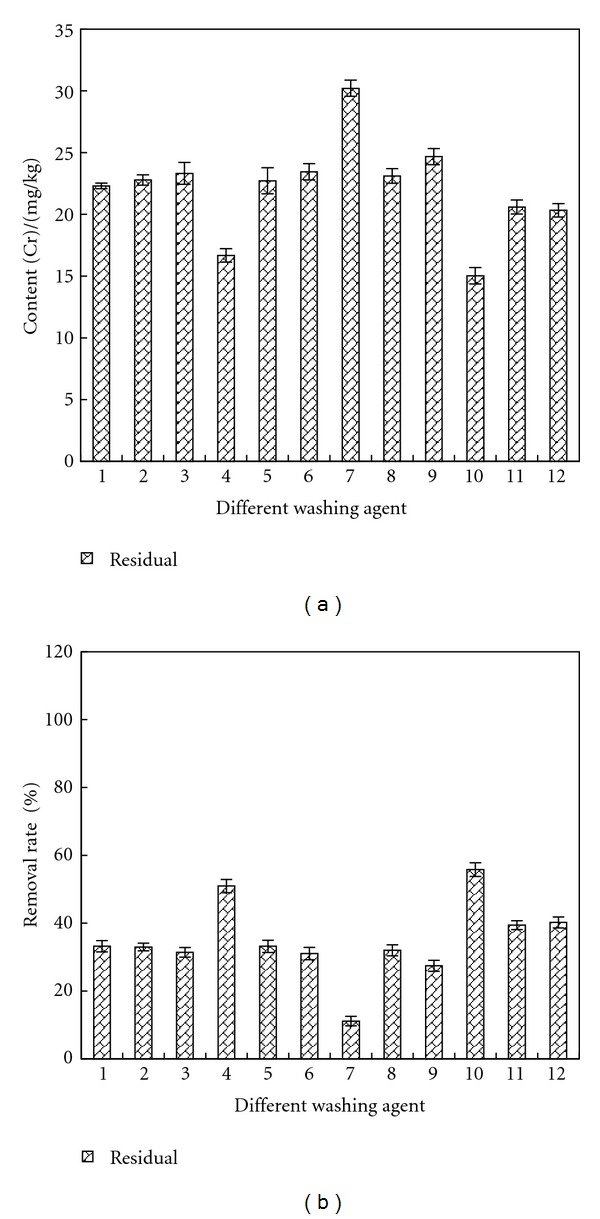
Removal effect of residual Cr by different washing agents. Washing agent: 1—water; 2—0.05 mol/L EDTA-Na_2_; 3—0.1 mol/L EDTA-Na_2_; 4—0.1 mol/L citric acid; 5—0.5 mol/L citric acid; 6—0.1 mol/L oxalic acid; 7—0.5 mol/L oxalic acid; 8—0.1 mol/L HCl; 9—0.5 mol/L HCl; 10—1 mol/L HCl; 11—0.1 mol/L acetic acid; 12—0.5 mol/L acetic acid.

Figure 11 shows the remained contents and removal rates of residual Cr in sample after being washed by different agents. As indicated by the results, the removal effects of all agents were not ideal and after disposal, the remained content of residual Cr was about 20–30 mg/kg. The removal rate was 30–50%.

## 4. Conclusions

1. Particle size had no distinct impact on the contents of total Cr and Cr (VI) in samples. Therefore, in the actual disposal, it shall select more economic crushing equipments regardless of the impact of particle size.
2. Although the removal rate of total Cr and Cr (VI) was about 75% after water washing, the content of Cr in sample was still high, and it was still quite harmful and needed further disposal by second washing.
3. Soluble Cr and reducible Cr were mainly made up of Cr (VI) and the reason that citric acid can obtain better removal effect is that they not only can dissolve the soluble Cr, but can reduce it into Cr (III) of other forms due to the redox.
4. Oxidizable Cr was mainly made up of Cr (III). Due to complexing action, EDTA, citric acid, oxalic acid, and acetic acid can better remove oxidizable Cr (III), especially citric acid.
5. After water washing, citric acid was used for it can better remove soluble Cr, acid soluble Cr, reducible Cr, and oxidizable Cr, and the removal rate of Cr (VI) was over 99% after disposal. The remained content of Cr (VI) in sample was lower than 10 mg/kg.

## Figures and Tables

**Table 1:** Physicochemical properties of the sample.

pH	*w* (total Cr)/(mg/kg)	*w* [Cr (VI)]/(mg/kg)	*w* (Cr)/(mg/kg)				
			Soluble	Acid soluble	Reducible	Oxidizable	Residual
11.91 ± 0.01	6714.67 ± 101.59	6387.96 ± 67.33	5164.80 ± 89.39	912.05 ± 5.71	250.02 ± 8.05	305.60 ± 8.45	126.06 ± 3.92

**Table 2:** Physicochemical properties of the sample after water washing.

pH	*w* (total Cr)/(mg/kg)	*w* [Cr (VI)]/(mg/kg)	*w* (Cr)/(mg/kg)				
			Soluble	Acid soluble	Reducible	Oxidizable	Residual
11.74 ± 0.04	1662.25 ± 65.45	1431.40 ± 41.29	1072.85 ± 54.65	347.25 ± 11.26	187.24 ± 4.56	156.68 ± 8.69	34.01 ± 5.93
